# Comparative analysis of full-length 16s ribosomal RNA genome sequencing in human fecal samples using primer sets with different degrees of degeneracy

**DOI:** 10.3389/fgene.2023.1213829

**Published:** 2023-07-26

**Authors:** Christian Waechter, Leon Fehse, Marius Welzel, Dominik Heider, Lek Babalija, Juan Cheko, Julian Mueller, Jochen Pöling, Thomas Braun, Sabine Pankuweit, Eberhard Weihe, Ralf Kinscherf, Bernhard Schieffer, Ulrich Luesebrink, Muhidien Soufi, Volker Ruppert

**Affiliations:** ^1^ Department of Cardiology, University Hospital Marburg, Philipps University Marburg, Marburg, Germany; ^2^ Max Planck Institute for Heart and Lung Research, Bad Nauheim, Germany; ^3^ Department of Mathematics and Computer Science, Philipps University Marburg, Marburg, Germany; ^4^ Institute of Anatomy and Cell Biology, Medical Faculty, Philipps University Marburg, Marburg, Germany; ^5^ Center for Undiagnosed and Rare Diseases, University Hospital Marburg, Philipps University Marburg, Marburg, Germany

**Keywords:** 16S rRNA, gut microbiome, human fecal microbiome, next-generation sequencing (NGS), nanopore sequencing, oxford nanopore technologies (ONT), MinION Mk1C, primer degeneracy

## Abstract

Next-generation sequencing has revolutionized the field of microbiology research and greatly expanded our knowledge of complex bacterial communities. Nanopore sequencing provides distinct advantages, combining cost-effectiveness, ease of use, high throughput, and high taxonomic resolution through its ability to process long amplicons, such as the entire 16s rRNA genome. We examine the performance of the conventional 27F primer (27F-I) included in the 16S Barcoding Kit distributed by Oxford Nanopore Technologies (ONT) and that of a more degenerate 27F primer (27F-II) in the context of highly complex bacterial communities in 73 human fecal samples. The results show striking differences in both taxonomic diversity and relative abundance of a substantial number of taxa between the two primer sets. Primer 27F-I reveals a significantly lower biodiversity and, for example, at the taxonomic level of the phyla, a dominance of *Firmicutes* and *Proteobacteria* as determined by relative abundances, as well as an unusually high ratio of *Firmicutes*/*Bacteriodetes* when compared to the more degenerate primer set (27F-II). Considering the findings in the context of the gut microbiomes common in Western industrial societies, as reported in the American Gut Project, the more degenerate primer set (27F-II) reflects the composition and diversity of the fecal microbiome significantly better than the 27F-I primer. This study provides a fundamentally relevant comparative analysis of the *in situ* performance of two primer sets designed for sequencing of the entire 16s rRNA genome and suggests that the more degenerate primer set (27F-II) should be preferred for nanopore sequencing-based analyses of the human fecal microbiome.

## Introduction

The study of complex bacterial communities associated with the human body, known as the microbiome, has experienced unprecedented growth over the past two decades and is currently one of the most intensively studied areas in biomedical science (Jones, 2013). The gut microbiome has been a particular focus of interest, as alterations in its complex and highly diverse composition are emerging as potential diagnostic biomarkers or pathogenetic factors for a plethora of disease (Gomaa, 2020). Accordingly, researchers, physicians and patients have high hopes for the further deciphering of microbial signatures and expect great therapeutic potential from specific modulation of the microbiome. The door-opener for this development has been the advent of next-generation sequencing (NGS) technologies, which has enabled a large number of laboratories to analyze complex microbiological communities by allowing rapid, accurate, and comparatively inexpensive sequencing of large amounts of DNA (Malla et al., 2019). The available sequencing platforms can be distinguished, for example, according to their ability to deliver different read lengths, and can thus be classified into short- and long-read technologies. The most widely used technology to date in microbiome research, including the American Gut Project and the Human Microbiome Project, is the Illumina platform, which delivers short reads with a maximum length of 2 × 300 base pairs (bp) using its latest version of the MiSeq^®^ system (Huttenhower et al., 2012; McDonalda et al., 2018; Ravi et al., 2018). As targeted sequencing of 16S ribosomal RNA (16s rRNA) gene has become the established method for amplicon-based identification of bacterial taxa in complex microbiological communities, one drawback of the Illumina technique has become apparent: Its short read length. The ∼1,500 bp bacterial 16S rRNA gene contains nine hypervariable regions (V1-V9) interrupted by highly conserved segments, which are suitable as anchor sequences for PCR primers. Illumina-based sequencing can therefore only target short fragments of the 16s rRNA gene, which in the majority of studies are amplified with primers targeting the V3 and V4 regions, which limits taxonomic resolution to the genus level, at best (Johnson et al., 2019; Matsuo et al., 2021; Szoboszlay et al., 2023). Third-generation sequencing platforms, such as the nanopore sequencing technology from Oxford Nanopore Technologies (ONT), have overcome these read-length limitations; the newest version of ONT’s nanopore sequencing provides an average read length of approximately 15 kbp and is thus easily capable of covering the entire 16s rRNA genome (Wang and Qian, 2009). Compared to the Illumina platform, however, the long read length comes at the expense of lower sequencing accuracy. Since the commercial launch of the ONT system in 2015, continuous improvements in device design, as well as its chemistry and bioinformatics, have reduced the initially very high error rate of about 6% down to well below 2% when using the very recently introduced nanopore sequencing kit Q20+ (LSK112) and the flow cell R10.4 (Delahaye and Nicolas, 2021; Luo et al., 2022). For comparison, Illumina’s error rate is between 0.1% and 1% (Stoler and Nekrutenko, 2021). However, even with this comparatively high error rate, the ONT system provides higher taxonomic resolution than short-read sequencing techniques due to its complete coverage of the 16s rRNA gene, which recent studies have shown extends down to the species level (Benítez-Páez et al., 2016; Shin et al., 2016; Nygaard et al., 2020; Matsuo et al., 2021). This impressive evolution of nanopore technology, combined with its convenient workflow and high cost-effectiveness render it an increasingly attractive and promising approach for the analysis of complex microbial communities, such as the human fecal microbiome. In this context, the selection of appropriate primer sets for 16s rRNA amplification is crucial, as it carries a major risk of biasing the detection of microbial signatures detected. Thus, in artificial microbial communities of known composition, it has been demonstrated that the selection of the 16s rRNA region can substantially influence the detected taxonomic diversity (Klindworth et al., 2013; Yang et al., 2016). In contrast, data on this topic are very limited for complex biological samples, especially for full-length 16s rRNA genome sequencing using the ONT platform. First results in this direction were provided by Matsuo et al. describing a primer-associated bias at the species level for *Bifidobacteria* in the context of 16s full-length rRNA sequencing with the nanopore technology (Matsuo et al., 2021). The present study elucidated the effects of primer selection on the microbial signature by systematically comparing the primers included in the very commonly used kit distributed by ONT with a primer set optimized according to the approach of Matsuo et al. (2021) in a large sample of complex human fecal samples.

## Materials and methods

### Sample collection and DNA extraction

Fecal samples from German donors without a history of relevant digestive tract disease such as chronic inflammatory bowel disease, cancers of the digestive tract or acute systemic or intestinal inflammation were collected using a special paper (#R1101-1-10, Zymo Research, Irvine, CA, United States) placed over the toilet seat to provide a low-germ environment and transferred into tubes containing DNA/RNA shielding buffer (#R1101, Zymo Research). After collection, samples were stored at room temperature and further processed within 3 days. Nucleic acid was extracted using the *Quick*-DNA^©^ HMW MagBead Kit (#D6060, Zymo Research) according to the manufacturer’s protocol. DNA purity and quantity were determined using NanoDrop^©^ (ThermoFisher Scientific, Waltham, MA, United States) and a Quantus^©^ Fluorometer (Promega, Madison, WI, United States), respectively, then stored at −20°C until further use.

### PCR amplification and nanopore 16s rRNA gene sequencing

From the DNA extracted as described above, two libraries were prepared, each with a different primer set.

For the construction of the first library (hereafter referred to as 27F-I library), 50 ng of whole genomic DNA was used and processed with the 16S barcoding kit containing the 16s rDNA primers 27F (5′- AGAGTTTGATC**M**TGGCTCAG -3′) and 1492R (5′- CGG​TTA​CCT​TGT​TAC​GAC​TT -3’; numbered according to the *Escherichia coli* rRNA; SQK-RAB204, ONT, Oxford, United Kingdom) according to the manufacturer’s protocol.

The second library (hereafter referred to as 27F-II library) was constructed using 50 ng of whole genomic DNA for the first PCR performed (see below) using a comparatively more degenerate 16s rDNA primer set [S-D-Bact-0008-c-S-20 and S-D-Bact-1492-a-A-22, (Klindworth et al., 2013; Matsuo et al., 2021)] with the anchor sequence 5′-TTTCTGTTGGTGCTGATATTGCAG**R**GTT**Y**GAT**YM**TGGCTCAG-3′ plus its reverse primer and with the anchor sequence 5′-ACTTGCCTGTCGCTCTATCTTCCGG**Y**TACCTTGTTACGACTT-3′ plus an appended barcode PCR according to the ONT protocol “Ligation sequencing amplicons - PCR barcoding (SQK-LSK110 with EXP-PBC096)”:

1. Preparation of 16s-PCR: 50 ng DNA in 11.5 µL nuclease-free water, 0.5 µL Primer 27F-II, 0.5 µL Primer1492R-II, 12.5 µL LongAMP^®^ Taq 2x Master Mix (New England Biolabs, Ipswich, MA, United States of America). Cycle program: 1 min 95°C; 25 cycles 20 s 95°C, 30 s 51°C, 2 min 65°C and a 5 min final elongation at 65°C.
2. Preparation of barcoding-PCR: 100 fmol 16S-PCR amplicons in 12.0 µL nuclease-free water, 0.5 µL barcode primer, 12.5 µL LongAMP^®^ Taq 2x Master Mix. Cycle program: 1 min 95°C; 15 cycles 20 s 95°C, 30 s 62°C, 2 min 65°C and a 5 min final elongation at 65°C.

After barcoding PCR, the DNA content of each amplicon was determined using a Quantus Fluorometer and adjusted to an equal volume. The amplicons were pooled, and 1 µg was used for library preparation. The library preparation was performed according to the protocol “Ligation sequencing amplicons–PCR barcoding (SQK-LSK110 with EXP-PBC096)” by ONT.

The bold characters in the primer sequences above indicate the degenerate bases, according to the code of the International Union of Biochemistry (IUB). This results in three different sequences for the 27F-I primer approach and 18 for the 27F-II primer approach (16 for the forward primer, 2 for the reverse primer). All sequence variants of the two primer approaches are listed in Supplementary Table S1.

The bar-coded libraries (27F-I and 27F-II libraries) were loaded and subsequently each sequenced on a separate flow cell (FLO-MIN106D, type R.9.4.1, ONT) using the MinION Mk1C device (ONT). MinKNOW version 22.03.4 (ONT) and Guppy 6.0.7 were used for data acquisition. Both libraries were prepared from DNA obtained by the same extraction procedure.

A total of 1,328,830 reads were generated for the library using the 27F-I primer approach (mean 18,203 reads, SEM 1,201 reads) and 1,578,822 reads were generated for the library using the 27F-II primer approach (mean 21,628 reads, SEM 1,991 reads, *p* = 0.14).

### Bioinformatics processing and analysis

Raw data processing was carried out with the Nanopore branch of Natrix (Welzel et al., 2020). Natrix is a modular sequencing read processing pipeline written in the workflow management engine Snakemake (Köster and Rahmann, 2012). The pipeline contains rules for the demultiplexing of raw sequencing data, quality control, removal of additional subsequences such as primer or barcodes, read assembly, dereplication, chimera detection and removal, abundance filtering, identification of representative sequences (either operational taxonomic units (OTU) or amplicon sequence variants), taxonomic assignment and additional assignment of meta information (for example, their functional roles or common habitats). A typical workflow used for this study is provided in the Supplementary Figure S1). Natrix processes a set of three file groups: A configuration file with user-configurable parameters (the choice of parameters used in this study are available in Supplementary Presentation S1), a primer table, containing information of additional subsequences for each sample, and the raw sequence files in FASTQ format. Natrix supports the filtering out of sequences lower than a user-defined quality score. For the initial quality filtering in this study, we used PRINSEQ (Schmieder and Edwards, 2011) with a mean quality threshold of 15, which corresponds to a maximum mean probability of a wrongly called base of around three percent. Every sequence read, that had a lower mean quality value below 15, was removed from further processing. We chose a more stringent quality threshold then the commonly used thresholds of 7–10 (Delahaye and Nicolas, 2021; Lee et al., 2021) for Nanopore data to reduce the probability of erroneous reads distorting the downstream analysis.

The removal of primers was carried out using a customized Porechop (https://github.com/rrwick/Porechop) version (https://github.com/MW55/Porechop). Porechop is a tool for the removal of adapter sequences in Nanopore reads. While the original Porechop version only searches for a hardcoded set of commonly used primers and can only remove a fixed number of bases from the end of a read, the customized version allows the definition of the primers by the user, and the removal of a fixed number of bases from both ends of the read. The minimal read length for a read to not be discarded by Porechop was set to 1,000 bases, while the maximal read length was set to 2000 bases. Additionally, Porechop was used to remove the first 100 bases from both ends to account for the decrease in read quality of Nanopore reads at both ends (Delahaye and Nicolas, 2021).

As the reads generated in this study were not paired end, no read assembly was carried out. The dereplication was carried out using the CD-HIT-EST algorithm (Fu et al., 2012) with a identity threshold of 1, to only combine reads that are 100% identical. The chimera detection utilized the uchime3_*denovo* algorithm of VSEARCH (Rognes et al., 2016), with the parameters beta 8.0, abskew 16 and pseudo_count 1.2. Natrix supports both the generation of OTUs and ASVs, but, as the ASV generation is carried out using DADA2, which uses a statistical model of Illumina error profiles (Callahan et al., 2016), the OTU modules of the pipeline were chosen for the generation of sequence clusters. OTUs were identified using VSEARCH (Rognes et al., 2016), using a similarity threshold of 85% and a minimal cluster size of 10 sequences. Compared to the more stringend 97% similarity threshold commonly used for OTU generation (Welzel et al., 2020), the lower similarity threshold was chosen to account for the increased error rates of Nanopore sequencing, compared to Illumina sequencing. Taxonomic information was assigned to the OTUs using the National Center for Biotechnology Information (NCBI) nucleotide (nt) database (Sayers et al., 2021) (latest as of 06/22), that contains sequences from Genbank (Benson et al., 2013), Refseq (O’Leary et al., 2016), TPA (Benson et al., 2015) and PDB (Berman et al., 2000), with the use of the nucleotide-nucleotide BLAST (BLASTn) algorithm of the BLAST+ (Camacho et al., 2009) toolkit. The taxonomic assignment was performed using a minimal identity overlap between target and query sequence of 90% and an E-value threshold of 10^–51^. The parameter max_target_seqs, which corresponds to the amount of hits per query that are returned by BLAST (Shah et al., 2018), was set to 10, with a subsequent filtering step that assigned the target sequence with the highest percentage identity times the logarithm of the alignment length to the query sequence.

### Statistical analyses

All statistical analyses and visualizations were performed using either the statistical programming language R with the *microeco* package (Liu et al., 2020), or the programming language Python. To compare the taxonomic composition via relative abundance on genus level between the two datasets acquired by sequencing using the two different primer sets 27F-I and 27F-II, a Pearson’s correlation test was performed. For the further statistical comparisons between the two datasets, the relative abundance on all different taxonomic levels, as well as the results for the alpha biodiversity measured via a Shannon Index, Wilcoxon singed-rank tests were performed and resulting p-values were corrected with the Benjamini-Hochberg method. All statistical tests performed accounted for the nature of paired analyses. A two-tailed p-value <0.05 was considered statistically significant.

## Results

Using a full-length 16S rRNA gene amplicon sequencing approach on the nanopore platform, we investigated the performance of the conventional 27F primer (hereafter referred to as 27F-I) included in the 16S Barcoding Kit (SQK-16S024) distributed by ONT, and that of a more degenerate 27F primer (hereafter referred to as 27F-II) covering possible polymorphisms in the conserved regions of the 16s rRNA genome in the context of highly complex bacterial communities derived from 73 human fecal samples. The comparative primer approach used is based on the four-primer PCR method described by Matsuo et al. (Klindworth et al., 2013; Matsuo et al., 2021), consisting of a PCR step with a more degenerate 27F and 1492R primer pair (S-D-Bact-0008-c-S-20 and S-D-Bact-1492-a-A-22, (Klindworth et al., 2013)) followed by barcoding PCR. OTUs were generated from the classifiable reads of the respective primer sets via alignment with the NCBI reference database and systematically compared.

**FIGURE 1:**
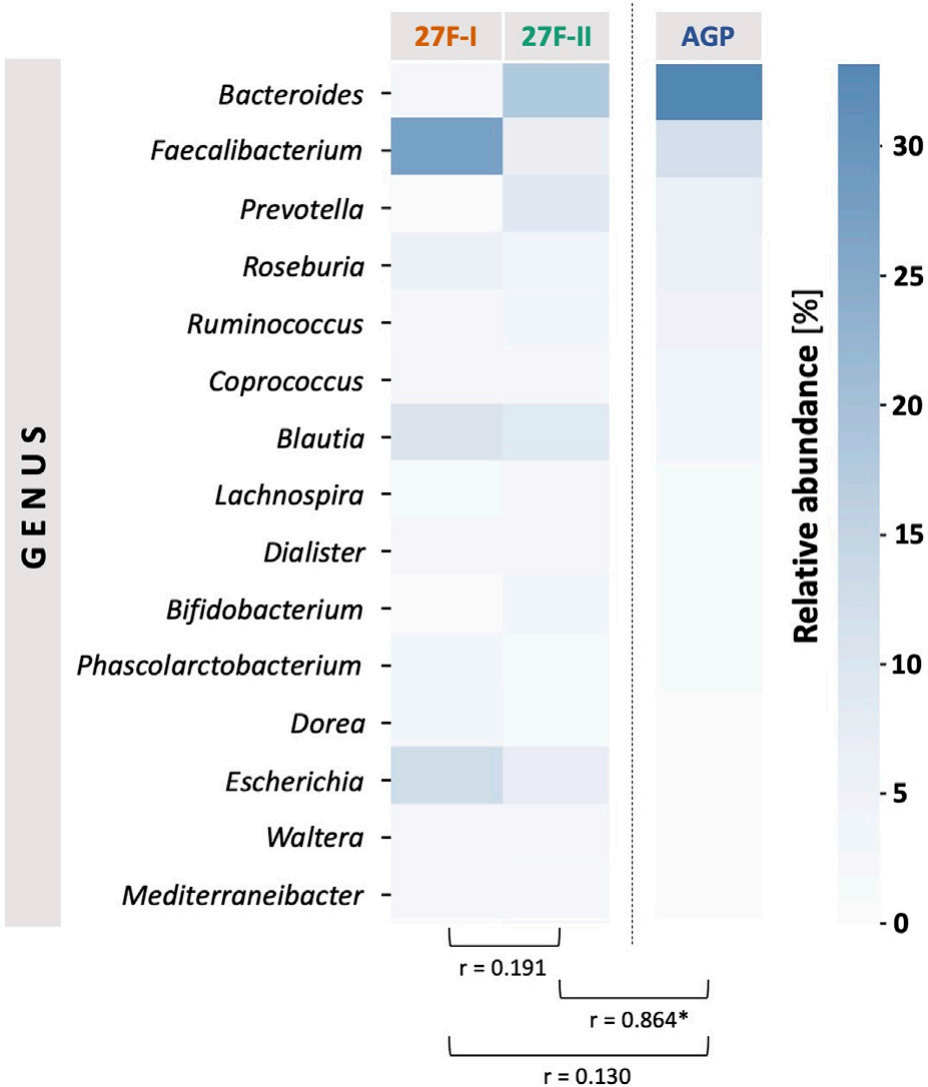
Comparison of the mean values of relative genus abundance for the 15 most abundant taxa across samples between the two primer sets using a heatmap and Pearson’s correlation (*r*). The American Gut Project (AGP) dataset of samples from individuals without intestinal disease was referenced for a normal taxonomic profile of a human fecal sample. The asterisk indicates statistical significance.

**FIGURE 2:**
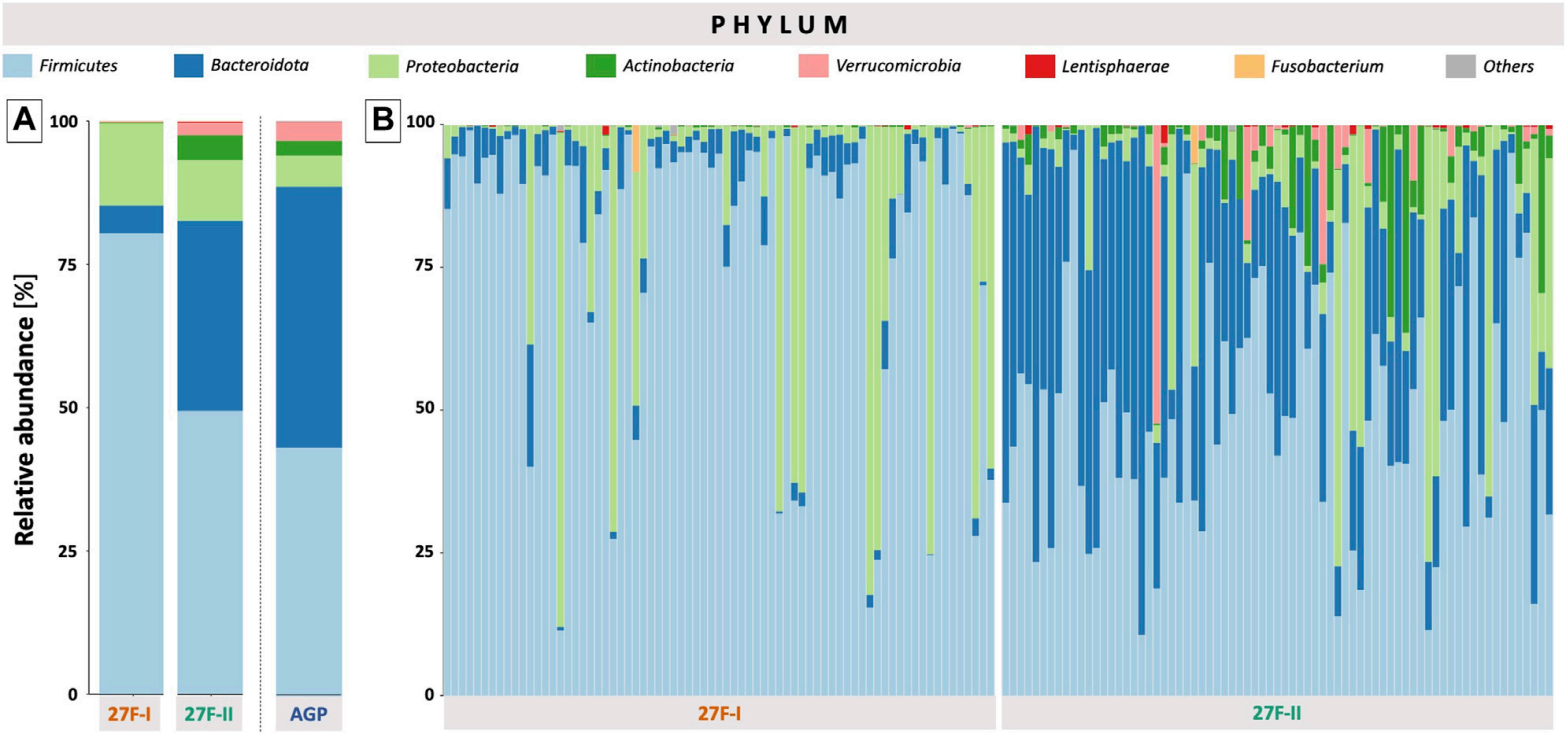
Overview of the relative abundance of the different phyla averaged over all samples **(A)** and at the individual sample level **(B)**. In Panel **(A)**, the American Gut Project (AGP) dataset of samples from individuals without intestinal disease was used as a reference for a normal phyla-level taxonomic profile of a human fecal sample.

**FIGURE 3:**
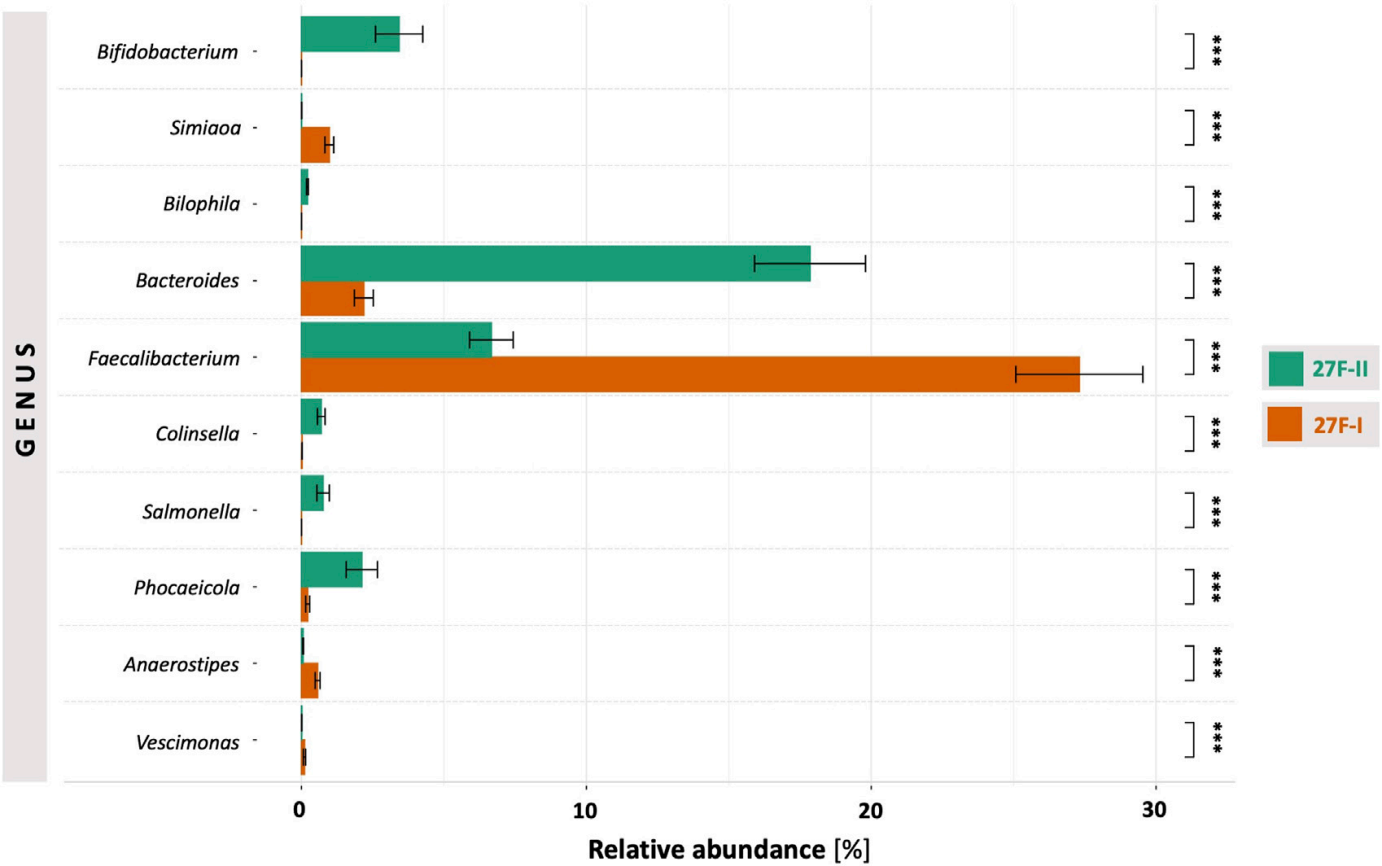
Comparison of genera with the most significant differences in abundance between the two primer approaches. ***–p-value < 0.001.

For a global comparison of the taxonomic profiles of human gut microbiota between the two primer sets, the Pearson correlation coefficient (*r*) was computed based on the mean values for the relative genera abundances across the samples for each primer approach. This revealed only a weak, statistically insignificant correlation (*r* = 0.191, *p* = 0.495) between the genera determined for the respective primer sets. To provide an estimate of which of the two primers more accurately maps the fecal microbiome, the taxonomic data generated by primers 27F-I and 27F-II were compared to an American Gut Project (AGP) dataset containing 3,560 samples from subjects without intestinal disease (McDonalda et al., 2018). This showed a statistically significant correlation between the taxonomic profile of fecal samples generated with primer 27F-II and the AGP dataset (*r* = 0.864, *p* = 3.29e-05). In contrast, there was only a weak, statistically insignificant correlation between the taxonomic profiles of the fecal samples generated with primer 27F-I and the AGP dataset (*r* = 0.130, *p* = 0.638). Figure 1 illustrates the comparison of relative genus abundance for the 15 most abundant taxa between the two primer sets using a heatmap. On further consideration, a clear discrepancy in the relative abundance is already evident at the taxonomic level of the phyla. The mean of all analyzed samples shows that the use of primer 27F-I results in a significantly higher abundance of *Firmicutes* (80.4% vs. 49.4%, *p* < 0.001) and a lower abundance of *Bacteroidota* (4.8% vs. 33.2%, *p* < 0.001), *Actinobacteria* (0.1% vs. 4.3%, *p* < 0.001), *Verrucomicrobia* (0.01% vs. 2.2%, *p* < 0.001) compared to primer 27F-II. Consequently, this leads to a significant divergence in the *Firmicutes/Bacteroidota* ratio (16.7 vs. 1.5, *p* < 0.001), discussed as a marker for dysbiosis (Ley et al., 2006), between the two primer sets. Figure 2 provides an overview of the relative abundance of the different phyla averaged over all samples and at the individual sample level. Supplementary Table S2 reports quantitative data on all bacterial phyla for the two sets of primers. At the taxonomic level of genera, statistically significant differences in relative abundance can be observed for a total of 125 distinct genera. When restricted to the ten genera with the most significant differences in abundance, the use of primer 27F-I results in a higher abundance of *Faecalibacterium* (7.306% vs. 6.666%, *p* < 0.001), *Simiaoa* (0.977% vs. 0.003%, *p* < 0.001), *Anaerostipes* (0.565% vs. 0.057%, *p* < 0.001) and *Vescimonas* (0.106% vs. 0.005%, *p* < 0.001) and a lower abundance of *Bacteroides* (2.189% vs. 17.853%, *p* < 0.001), *Bifidobacterium* (0.000% vs. 3.427%, *p* < 0.001), *Phocaeicola* (0.216% vs. 2.118%, *p* < 0.001), *Salmonella* (0.000% vs. 0.759%, *p* < 0.001), *Clinsella* (0.021% vs. 0.693%, *p* < 0.001) and *Bilophila* (0.000% vs. 0.210%, *p* < 0.001) compared to primer 27F-II as shown in Figure 3. Complete quantitative data for all genera are provided in Supplementary Table S3. As the 16S Barcoding Kit (SQK-16S024) containing primer 27F-I has only been validated for genera-level resolution, species-level resolution was not performed.

**FIGURE 4:**
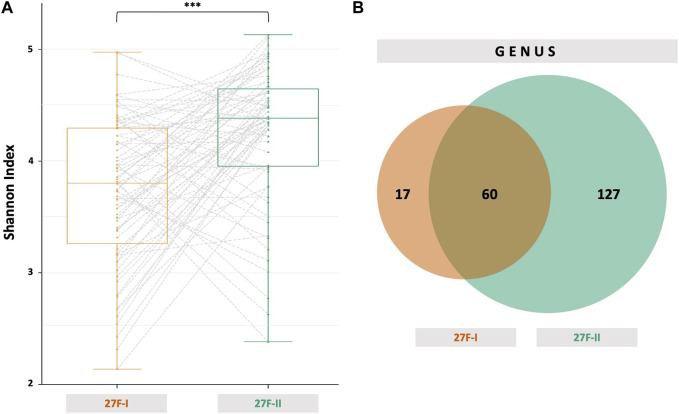
Alpha diversity represented as Shannon index **(A)** for the two primer approaches and a Venn diagram **(B)** showing the common and specific taxonomic units at the genus level between the two primer sets used. The dashed gray lines in **(A)** link the results of a sample after analysis with 27F-I and 27F-II primer set, respectively. ***–p-value < 0.001.

In addition to the outlined discrepancies in the taxonomic profiles of the human gut microbiome, significant differences in taxonomic diversity are also apparent, depending on the primer set used. Primer 27F-I detects notably fewer distinct OTUs in the human fecal samples than primer set 27F-II, as reflected by a statistically significant lower Shannon index (3.733 vs. 4.271, *p* < 0.001, Figure 4) expressing alpha diversity.

## Discussion

The introduction of next-generation sequencing has revolutionized the field of microbiology research and greatly expanded our knowledge of complex human enteric bacterial communities. In this context, nanopore sequencing stands out as combining the advantages of cost-effectiveness, simplicity of use, high throughput, and high taxonomic resolution through its ability to read long amplicons. Recent substantial advances in sequencing accuracy significantly mitigate what has been a significant weakness of this technology and represent a culmination of this impressively rapid technical evolution of the nanopore platform, which has allowed it to eclipse the performance of short-read sequencing techniques. Not least, the 16S Barcoding Kit (SQK-16S024) offered by ONT and widely used in the community contributes notably to the simplicity, speed, and high cost-effectiveness of nanopore 16S rRNA gene sequencing (Santos et al., 2020).

As the choice of primers is known to have a decisive impact on qualitative and quantitative taxonomic signatures (Armougom, 2009), we tested the performance of the primer set included in the commercial 16S Barcoding Kit (referred to here as 27F-I) and compared it to a more degenerate primer set (referred to here as 27F-II) on complex microbial communities. Our analyses demonstrate striking differences in both taxonomic diversity and relative abundance of a high number of different taxa between the two primer approaches in a large sample of human fecal specimens: The primer set included in the commercial kit (27F-I) results in significantly lower bacterial biodiversity, as measured by the Shannon index and, for example, at the taxonomic level of phyla, a dominance of *Firmicutes* and *Proteobacteria* as measured by relative abundances as well as an unusually high ratio of *Firmicutes/Bacteriodetes* compared to the degenerate primer set (27F-II). These substantial differences in relative abundances of taxa are detectable at all taxonomic levels and result, for example, in a lower relative abundance of the genera *Bacteriodes, Bifidobacterium* and *Phocaeicola* and a higher relative abundance of *Faecalibacterium* when using primer 27F-I compared to using primer 27F-II. Also, Pearson’s correlation shows only a weak, statistically non-significant correlation between the taxonomic signatures generated by the two primer sets. Comparing our microbiome data with commonly observed fecal microbiome signatures in Western industrial societies, such as those derived from the American Gut Project (AGP), indicates that the 27F-II primer more reliably reflects the fecal microbial composition and diversity than the primer 27F-I (Huttenhower et al., 2012). Despite a comparable Western lifestyle and level of urbanization of our population and the AGP population, as well as a sequencing approach that is also 16s rRNA gene amplicon-based targeting V4 region, such a comparison can only be indicative and is subject to several limitations. Apart from the fundamentally different sampling, both sample collection and DNA extraction were performed according to different protocols. The main difference between the AGP dataset and the present data is the use of the Illumina short-read sequencing platform with all its differences, e.g., selection of the conserved region of the 16s rRNA genome for amplification, choice of corresponding primer sets, and the subsequent bioinformatic processing.

Analysis of the 27F primer binding sites by Frank et al. may explain the differences between the 27F-I and 27F-II approaches. The commonly used 27F primer formulations, including the 27F-I primer set, do not cover several sequence variations involving contiguous phylogenetic clusters (Frank et al., 2008). The exclusion of such sequence variations explains the striking underrepresentation of several essential phylogenetic groups when the 27F-I primers or the “standards”, e.g., from the AGP or Human Microbiome Project (HMP), are used. For particular taxa such as *Bifidobacterium* or *Bacteroides,* several base mismatches with the 27F-I forward primer were identified, consistent with the comparative underrepresentation of these genera in the samples analyzed with primer 27F-I (Frank et al., 2008; Matsuo et al., 2021). In contrast, the optimal coverage of the taxon *Faecalibacterium* by the 27F-I primer would explain its higher relative abundance compared to the 27F-II based analysis. Another potential, albeit unlikely, explanation for the reduced relative abundance of the taxon *Faecalibacterium* in the 27F-II primer compared to the 27F-I primer approach may involve dilution effects reported when using degenerate primers (Linhart and Shamir, 2005; Frank et al., 2008).

In addition to the more faithful representation of taxonomic abundancies, the higher degree of degeneracy of the 27F-II primer allows superior mapping of fecal microbiome diversity compared to 27F-I primers. This is a particular advantage when analyzing complex microbial samples, which have very high genetic diversity, requiring amplification of numerous unknown target sequences (Frank et al., 2008; Klindworth et al., 2013).

The disadvantage of using a degenerate primer is the greater potential for non-specific amplification or primer dimer formation, which can reduce PCR efficiency and accuracy (Dieffenbach et al., 1993). However, this problem can be effectively addressed by appropriate processing of sequenced reads, as the length of the expected amplicon can be accurately predicted and accounted for in the filter settings.

## Conclusion

Recent advances in sequencing chemistry and base-calling algorithms have improved accuracy of ONT, which provides higher taxonomic resolution of full-length 16S rRNA gene sequencing compared to short-read sequencing (Shin et al., 2016; Johnson et al., 2019; Wei et al., 2020; Matsuo et al., 2021). It is highly likely that the time- and cost-efficient Nanopore platform will play an increasingly important role in the expanding field of microbiome research in the future. Our study provides a relevant comparative analysis of the performance of two different primer sets designed for full 16s rRNA genome sequencing of complex *in situ* samples. We demonstrate limitations of the universal 27F primer set (here referred to as 27F-I) for reliable detection of microbiome signatures in complex samples, such as human feces. In contrast, the more degenerate 27-F primer set (here referred to as 27F-II) uncovers microbial signatures much more faithfully and should be preferred for nanopore sequencing-based analyses of the human fecal microbiome.

The present study provides novel and important implications for both scientific and clinical applications when conducting microbial community analysis.

## Data Availability

The datasets presented in this study can be found in online repositories. The names of the repository/repositories and accession number(s) can be found in the article/Supplementary Material.
