# Supplementary material for: Comparative analysis of full-length 16s ribosomal RNA genome sequencing in human fecal samples using primer sets with different degrees of degeneracy
Source: Front Genet. 2023 Jul 26;14:1213829. doi: 10.3389/fgene.2023.1213829 (PMC10411958; doi:10.3389/fgene.2023.1213829)
Supplement: Supplementary file 1 [file Presentation1.pdf]

General options
general:
filename : waechter # The path / filename of the project folder, primertable (.csv) and configfile (.yaml). If the raw data folder is not in the root directory of Natrix, please add the path relative to the root directory (e.g. input/example_data)

primertable: waechter.csv # Path to the primertable. If the primertable is not in the root directory of Natrix, please add the path relative to the root directory (e.g. input/example_data.yaml)
units: units.tsv # Path to the sequencing unit sheet.
cores : 20 # Amount of cores available for the workflow.
multiqc : FALSE # Initial quality check (fastqc & multiqc), currently only works for not yet assembled reads.
demultiplexing: FALSE # Boolean, run demultiplexing for reads if they were not demultiplexed by the sequencing company (slow).
read_sorting: FALSE # Boolean, run read sorting for paired end reads if they were not sorted by the sequencing company (slow).
already_assembled: FALSE # Boolean, skip the quality control and read assembly steps for data if it is already assembled.
seq_rep: OTU # Type of sequence representative, possible values are: "ASV", amplicon sequence variants, created with DADA2 or "OTU", operational taxonomic units, created with SWARM
sequencing: Nanopore # Sequencing method used, 'Illumina' or 'Nanopore'.

Quality check and primer removal
qc:
threshold : 0.9 # PANDAseq score threshold a sequence must meet to be kept in the output.
minoverlap : 15 # Sets the minimum overlap between forward and reverse reads.
minqual : 1 # Minimal quality score for bases in an assembled read to be accepted by PANDAseq.
minlen : 1000 # The minimal length of a sequence after primer removal to be accepted by PANDAseq, Cutadapt, or Porechop.
maxlen : 2000 # The maximal length of a sequence after primer removal to be accepted by PANDAseq, Cutadapt, or Porechop.
head_crop: 100
tail_crop: 100
primer_offset : FALSE # Using PANDAseq to remove primer sequences by length offset instead of sequence identity, only for OTU variant.
mq : 15 # Minimum quality sequence check (prinseq), filtering of sequences according to the PHRED quality score before the assembly.
barcode_removed: TRUE # Boolean that indicates if the sequence is free of barcodes.
all_primer : FALSE # Boolean that indicates if the sequence is free of any kind of additional subsequences (primer, barcodes etc.).

Dereplication
derep:

clustering : 1 # Percent identity for cdhit (dereplication) (1 = 100%), if cdhit is solely to be used for dereplication (recommended), keep the default value.

length_overlap : 0.0 # Length difference cutoff, default 0.0 if set to 0.9, the shorter sequences need to be at least 90% length of the representative of the cluster.

representative: most_common # Which sequence to use as a representative sequence per CDHIT cluster. longest = the longest sequence of the corresponding cluster, most_common = the most common sequence of the corresponding cluster.

Chimera removal
chim:

beta : 8.0 # Weight of a "no" vote for the VSEARCH chimera detection algorithm.

pseudo_count : 1.2 # Pseudo - count prior on number of “no” votes.

abskew : 16 # Minimum abundance skew, definied by (min(abund.(paren1), abund.(paren2))) / abund.(child).

Merging
merge:

filter_method : not_split # If the split sample approach was used (split_sample) or not (not_split).

ampliconduo: FALSE # Boolean, whether AmpliconDuo should be used for statistical analysis of the data.

cutoff : 1 # An additional abundance filter if the split sample approach was not used.

ampli_corr : fdr # Specifies the correction method for Fisher's exact test.

save_format : png # File format for the frequency-frequency plot.

plot_AmpDuo : FALSE # Boolean, whether the frequency-frequency plot should be saved.

paired_End : FALSE # Boolean. Format of the sequencing data, TRUE if the reads are in paired-end format.

name_ext : R1 # The identifier for the forward read (for the reverse read the 1 is switched with 2, if the data is in paired-end format), has to be included at the end of the file name, before the file format identifier (including for single end files).

vsearch_clust_id: 0.85

vsearch_clust_min: 10

vsearch_clust_output: centroids

BLAST
blast:

blast : True # Boolean to indicate the use of the BLAST clustering algorithm to assign taxonomic information to the OTUs.

database: NCBI # Database against which the BLAST should be carried out, at the moment "NCBI" and "SILVA" are supported.

drop_tax_classes: '' # given a comma-separated list, drops undesired classes either by id, by name or using regex

db_path : database/ncbi/nt # Path to the database file against which the BLAST should be carried out, at the moment only the SILVA (database/silva/silva.db) and NCBI (database/ncbi/nt) databases will be automatically downloaded.

max_target_seqs : 10 # Number of NCBI blast hits that are saved per sequence / OTU.

ident : 90.0 # Minimal identity overlap between target and query sequence. Set to lower threshold to be able to filter later by hand-

evalue : 1e-51 # Highest accepted evalue. Set to higher threshold to be able to filter later by hand.

split_filtered_blast_table : TRUE # Boolean if results of filtered_blast_table should additionally be splitted into files by taxonomic rank.

annotate_rank: Species # one of ['Phylum','Class','Order','Family','Genus','Species'] or null; only if ncbi; queries opus23 utopia search and extracts their tags
