# Supplementary figures and images for: Comparative analysis of full-length 16s ribosomal RNA genome sequencing in human fecal samples using primer sets with different degrees of degeneracy
Source: Front Genet. 2023 Jul 26;14:1213829. doi: 10.3389/fgene.2023.1213829 (PMC10411958; doi:10.3389/fgene.2023.1213829)

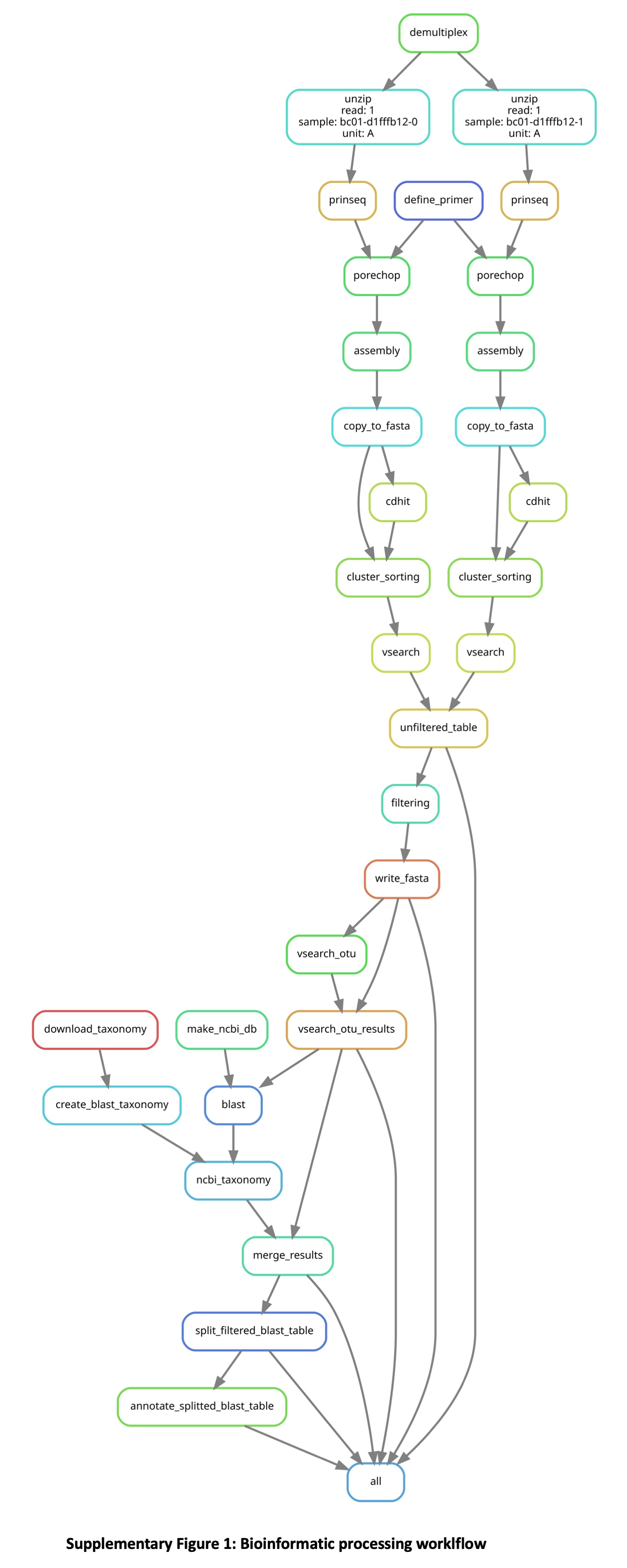

Supplement: Supplementary file 5 [file Image1.PNG]
